# Porphyrin Molecules Decorated on Metal-Organic Frameworks for Multi-Functional Biomedical Applications

**DOI:** 10.3390/biom11111714

**Published:** 2021-11-17

**Authors:** Navid Rabiee, Mohammad Rabiee, Soheil Sojdeh, Yousef Fatahi, Rassoul Dinarvand, Moein Safarkhani, Sepideh Ahmadi, Hossein Daneshgar, Fatemeh Radmanesh, Saeid Maghsoudi, Mojtaba Bagherzadeh, Rajender S. Varma, Ebrahim Mostafavi

**Affiliations:** 1 Department of Physics, Sharif University of Technology, Tehran 11155-9161, Iran; 2 School of Engineering, Macquarie University, Sydney, NSW 2109, Australia; 3 Biomaterial Group, Department of Biomedical Engineering, Amirkabir University of Technology, Tehran 15875-4413, Iran; mrabiee@aut.ac.ir; 4 School of Chemistry, College of Science, University of Tehran, Tehran 14155-6455, Iran; sojdesoheil@gmail.com; 5 Department of Pharmaceutical Nanotechnology, Faculty of Pharmacy, Tehran University of Medical Sciences, Tehran 14155-6451, Iran; youseffatahi@gmail.com (Y.F.); dinarvand@tums.ac.ir (R.D.); 6 Nanotechnology Research Center, Faculty of Pharmacy, Tehran University of Medical Sciences, Tehran 14155-6451, Iran; 7 Department of Chemistry, Sharif University of Technology, Tehran 11155-3516, Iran; moein.s.1991@gmail.com (M.S.); daneshgar.ho@gmail.com (H.D.); m.bagherzadeh.sharif@gmail.com (M.B.); 8 Student Research Committee, Department of Medical Biotechnology, School of Advanced Technologies in Medicine, Shahid Beheshti University of Medical Sciences, Tehran 19857-17443, Iran; speahmadi@yahoo.com; 9 Cellular and Molecular Biology Research Center, Shahid Beheshti University of Medical Sciences, Tehran 19857-17443, Iran; 10 Uro-Oncology Research Center, Tehran University of Medical Sciences, Tehran 14197-33141, Iran; rad.biotech89@gmail.com; 11 Faculty of Medicine, Department of Physiology and Pathophysiology, University of Manitoba, Winnipeg, MB R2H 0G1, Canada; maghsous@myumanitoba.ca; 12 Regional Centre of Advanced Technologies and Materials, Czech Advanced Technology and Research Institute, Palacky University, Šlechtitelů 27, 783 71 Olomouc, Czech Republic; 13 Stanford Cardiovascular Institute, Stanford University School of Medicine, Stanford, CA 94305, USA; 14 Department of Medicine, Stanford University School of Medicine, Stanford, CA 94305, USA

**Keywords:** gene delivery, CRISPR, biosensor, biomedicine, MOF, COVID-19

## Abstract

Metal–organic frameworks (MOFs) have been widely used as porous nanomaterials for different applications ranging from industrial to biomedicals. An unpredictable one-pot method is introduced to synthesize NH_2_-MIL-53 assisted by high-gravity in a greener media for the first time. Then, porphyrins were deployed to adorn the surface of MOF to increase the sensitivity of the prepared nanocomposite to the genetic materials and in-situ cellular protein structures. The hydrogen bond formation between genetic domains and the porphyrin’ nitrogen as well as the surface hydroxyl groups is equally probable and could be considered a milestone in chemical physics and physical chemistry for biomedical applications. In this context, the role of incorporating different forms of porphyrins, their relationship with the final surface morphology, and their drug/gene loading efficiency were investigated to provide a predictable pattern in regard to the previous works. The conceptual phenomenon was optimized to increase the interactions between the biomolecules and the substrate by reaching the limit of detection to 10 pM for the Anti-cas9 protein, 20 pM for the single-stranded DNA (ssDNA), below 10 pM for the single guide RNA (sgRNA) and also around 10 nM for recombinant SARS-CoV-2 spike antigen. Also, the MTT assay showed acceptable relative cell viability of more than 85% in most cases, even by increasing the dose of the prepared nanostructures.

## 1. Introduction

The study of biological properties along with chemical and physical optimizations on a molecular scale has been conducted by several techniques, and they have made significant advancements over time, leading to enhanced accuracy [1,2,3]. However, this enhancement has brought about a significant increase in cost and time, in particular for biomedical studies that are being conducted through expensive and high-precision techniques. In many cases, unfortunately, these features can stop this research from entering into the clinical phase. Therefore, it is imperative that special attention be paid to the maximum use of simple systems, but within the limits of acceptable standards [4,5,6,7,8,9,10,11,12]. So far, there have been very limited works that deal with sustainable chemistry and circular economy in their concepts, even in the biomedical sciences. This scientific area has reached a new phase of advancements; however, the cost of preparation of the pre-clinical and clinical materials causes these steps to be a luxury method. Therefore, scientists should focus more on using precisely sustainable chemistry and circular economy rules [13,14,15,16,17].

To date, in various fields of basic sciences, especially chemistry and physics, standards have been set for low-cost optimizations and early conclusions. However, in biomedical studies, no specific standards have yet been defined. The need for early decisions on methods, approaches, or even groups of synthetic chemical compounds deployed for biomedical applications makes tremendous sense. In the meantime, such standards can also help research advance more expeditiously and efficiently. Our aim in this study is to focus on establishing a physicochemical standard for smart cargo delivery in inorganic systems [18,19,20,21,22,23,24,25]. Also, interdisciplinary researchers could be considered a tool for rapid movements and advancements to the early diagnosis and detection of different kinds of diseases, pathogens, and biomarkers. One of the overlooked facts regarding the research in this field is the importance of rapid and early diagnosis as well as the detections through simple, green, and cost-effective methods. Therefore, one of the most important aspects of this work is to develop a nanosystem via a simple, environmentally friendly, and cost-effective method for highly advanced diagnosis and detection systems [26,27,28,29,30]. MOFs, due to their high porosity, surface functionalizations, and biodegradable bulk properties, as well as their different sized particles, channels, and pores, have gained scientists’ attention. In this regard, different forms of MOFs have been synthesized and characterized for different biomedical applications; however, using MOFs as a substrate would be a wise choice rather than using them as an active probe. Recently, we used UiO-66-based nanomaterials for co-delivery of a drug and a genetic material (Doxorubicin and pCRISPR) and showed that the surface of the MOF could be decorated with different types of biodegradable polymers as well as the leaf extracts. Therefore, it should be possible to form different types of nanocomposites with inorganic and organic components [31,32,33].

Many autoimmune diseases, and even genetic and acute infectious diseases, can be diagnosed by very small changes in the number of bioactive molecules in biological fluids, including blood. To this end, early detection of very small changes in the concentration and even the number of bioactive molecules in biological fluids can be very significant. In the case of cancer, there are substantial changes in vitamins and even natural proteins, which can be detected in the early stages. In the case of other acute diseases, these changes can lead to alterations in genetic structures and even in the presence of certain biomolecules. However, the main problem here is the early detection of the variations and associated biomolecules. Electrochemical sensors are now widely and routinely used for early detection. Nevertheless, the ease of operation, cost-effectiveness, simple optimization, and reliability of optical sensors are not comparable to electrochemical types. Therefore, the development of innovative technologies in optical sensors can give rise to the emergence of new and reliable methods. To date, no specific research has been conducted on the detection and identification of biomolecules and proteins having both selectivity and sensitivity towards all of the anti-cas9 protein, ssDNA, and sgRNA; conducted studies are based on the development of electrochemical methods that require special equipment, and are not easily developed to point-of-care devices [34,35,36,37,38,39,40,41,42]. In order to prepare an advanced sensitive nanomaterial with multi biofunctional applications, two crucial parameters should be taken into account. The first is to possess a suitable, highly porous biocompatible, and tunable substrate, which in this case, a MOF has been selected. The second aspect concerns using a highly sensitive molecule as a sensitizer, for which a porphyrin has been selected due to its considerable biocompatibility, quantum yield, and significant quantum lifetime [31,43,44,45].

This work aims to design, synthesize, and characterize a cost-effective and greener MOF and investigate their surface morphology and potential relationships with the biological properties. Further, with the objective of an early decision on the biomedical features of the synthesized nanocomposite, we exclusively focused on morphological analysis. The MOF was adorned with phthalocyanine-like molecules (porphyrin) to enhance the sensitivity of the nanomaterial towards different biomolecules. In this manner, field emission electron microscopy (FESEM), transmission electron microscopy (TEM), and atomic force microscopy (AFM) analysis were conducted, and the results were analyzed based on them. In the next step, the sensitivity of the synthesized nanomaterials in the presence of ssDNA, sgRNA, and Anti-cas9 protein was investigated through screening the fluorescence spectra of the surface decorated porphyrins. Also, the relative cell viability of the synthesized nanomaterials was investigated comprehensively via MTT assay on the HEK-293, HeLa, HepG2, and PC12 cell lines after 24 and 48 h of treatment.

## 2. Methods

### 2.1. Synthesis of NH2-MIL-53 via High-Gravity Technique

**Figure 1:**
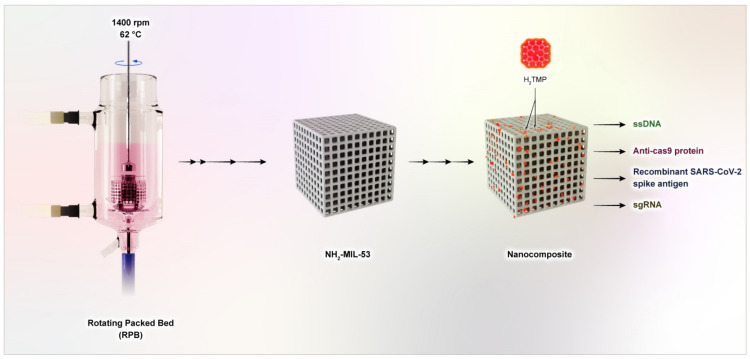
A schematic illustration of the synthesis and preparation of the MOF-based nanocomposite with the assistance of a high-gravity technique.

The synthesis concept for NH_2_-MIL-53 is based on the preceding literature [46,47]. However, in this work, a new synthesis procedure is developed with the assistance of a high-gravity technique and deploying the green chemistry approach adapted from our previous works [21,48,49]. Briefly (as depicted in Figure 1), 2.3 mmol of CrCl_3_.6H_2_O (Sigma-Aldrich, Germany) and 2.3 mmol of 2-aminoterphtalic acid (H_2_BDC-NH_2_) (Sigma-Aldrich, Germany) were mixed and transferred to a 100 mL jar, and the mixture was dissolved by the addition of 85 mL of water/ethanol (1:1) and 1 mL of formic acid (Sigma-Aldrich, Germany). In this step, the modified rotating packed bed (RPB) system was applied as described in our previous articles [50], which has a sealed ring and a packed rotator, and a jacket for temperature control, as well as the necessary inlets and outlets. For the synthesis procedure, the solution mixture was transferred to the internal circulation space of the RPB system through the inlet. The rotation of this RPB system was adjusted to 1400 rpm, which resulted in a high-gravity factor of 182. The temperature of the RPB system was adjusted to 62 °C (that is more than 10 °C below the typical reaction protocols [51,52]), and the internal space of the RPB was degassed by flowing the oxygen for 1.5 h prior to starting the system. After about 1.5 h (which is also 2 h below the typical reaction [51,53]), the samples were cooled down to room temperature under the gas flow of NH_3_, and after that, the central system of RPB was degassed with the N_2_ prior to collecting the synthesized nanomaterial. Then, the synthesized MOF was placed in a solution containing 1.5 g of *Rosmarinus officinalis* (leaf extracts) to modify the surface; the suspension was stirred for 6 h at room temperature.

### 2.2. Fabrication of Nanomaterial for Biomedical Assay

In order to prepare the suitable nanomaterial for biomedical assay, the synthesized H_2_TMP (3 mg) was dispersed into DMF (10 mL) and sonicated in a dark place for 17 min, and then the modified MOF (8 mg) was added to the mixture. The final mixture was stirred for 24 h in a dark place at room temperature.

### 2.3. Cytotoxicity Analysis

First, all of the materials and nanomaterials were sterilized using ultraviolet exposure. Then, in order to assess the relative cell viability, a cytocompatibility assessment was conducted using MTT (3-[4,5-dimethylthiazol-2-yl]-2,5-diphenyltetrazolium bromide) (MTT, Sigma, Germany) colorimetric assay at three different time points of 24, 48, and 72 h. In this regard, four kinds of cell lines, including PC12 cells (ATCC CRL-1721^TM^), HEK-293 (ATCC CRL-1721^TM^), HeLa (ATCC CCL-2), and HepG2 (ATCC HB 8065), were applied. Briefly, 1 × 10^5^ cells per well were cultured on the substrate in Dulbecco’s Modified Eagle’s Medium (DMEM, Gibco, Germany) containing 100 IU/mL streptomycin and 100 IU/mL penicillin (Invitrogen, Germany), and 10% fetal bovine serum (FBS; Gibco, Germany), and then, the resultants were incubated at the normal condition (37 °C at 5% CO_2_). After that, 100 μL of the MTT solution (5 mg/mL in PBS) was added to each well, and then, the medium was replaced with the formazone precipitates (dissolved in dimethyl sulfoxide (DMSO; Sigma-Aldrich, Germany). The optical absorbance was measured at 570 nm using a microplate Elisa reader (ELX808, BioTek, Germany).

### 2.4. Investigation of the ssDNA, sgRNA, Anti-cas9 Protein, and Recombinant SARS-CoV-2 Spike Antigen Interaction with the Modified Nanocomposite

In order to analyze the possible interaction between the ssDNA (the sequence of the used ssDNA was 5′-SH-(CH_2_)_6_-TGT GGG GGT GGA CGG GCC GGG TAGA-3′), sgRNA, Anti-cas9 protein, and recombinant SARS-CoV-2 spike antigen and the synthesized MOF, 100 µL of the MOF-porphyrin (with the concentration of 10 mg/mL) (as the number 1 concentration (main concentration)) and 40 µL of analyte (as the number 1 concentration (main concentration)) were incubated for 30 min at 3 °C. Different concentrations of the prepared host–guest molecules were prepared as depicted in the main text image. These complexes were purified by centrifuging at 14,000 rpm for 10 min. In the next step, the precipitates were dissolved in ultrapure water and washed with an excess amount of water.

## 3. Results and Discussion

**Figure 2:**
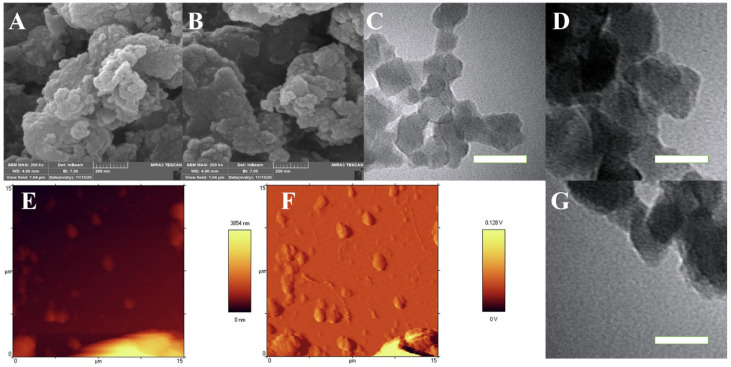
FESEM (**A**,**B**) (from different places to prove the homogeneity of the nanostructure), TEM (**C**,**D**,**G**) (from different places to prove the homogeneity of the nanostructure), and 2D AFM (**E**,**F**) (from different places to prove the homogeneity of the nanostructure) results of the NH2-MIL-53@H2TMP. Scale bar of the TEM images is 80 nm.

**Figure 3:**
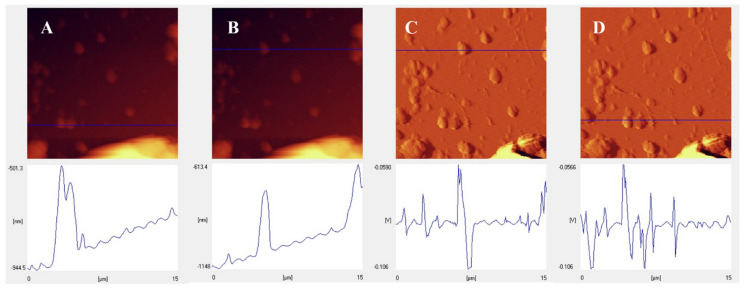
Line analysis roughness with different positions (**A**–**D**) images of the NH_2_-MIL-53@H_2_TMP.

As mentioned in the introduction, the aim of this project is to establish a standard for predicting the biological properties through minimal identification of synthesized nanomaterials. For this purpose, in this project, only surface characterizations were used to accurately understand the surface morphology. The FESEM images (Figure 2A,B) show that the cubic and semi-cubic nanostructures that stand for the MOF appeared with lots of aggregations that originated from the presence of leaf extracts. In this study, the aim was to use a MOF-based nanostructure without any extensive purifications, synthesized with the assistance of green media for biomedical applications. Therefore, these aggregations were not detected as a negative result for this kind of study. The TEM images (Figure 2C,D,G) showed the cubic nanostructural morphology of the MOF clearly, with a sharp natural-based coating on the edges of these MOF’s. The size of the MOF is in the range of 40 to 70 nm, which is suitable for biomedical applications. The results of the AFM investigations (Figure 2E,F) showed aggregations and agglomerations in some of the sites, but most of the surfaces were free of any chemical and/or physical molecular stackings. Also, the roughness of these MOF-based surfaces (Figure 3A–D) is not homogenized and could result in the physical interaction between different biomolecules and genetic materials with these surfaces. These physical interactions could happen through the small protein-based domains in the biomolecules and the roughness of more than 8 nm, but for some of the genetic materials, these interactions in the presence of roughness more than 5 nm have been observed as well [54,55,56]. To ascertain the successful synthesis of the MOF’s, the powder X-ray diffraction (PXRD) and Fourier transform infrared spectroscopy (FTIR) was performed (Supplementary Materials Figures S1 and S2), and the results were compared to the literature, which affirmed the successful synthesis of the MOF. Additionally, appropriate elemental mapping of the synthesized MOF assisted by high-gravity and the deployment of leaf extracts was conducted. The results (Supplementary Materials Figure S3) indicated the presence of the main elements on the surface of the green synthesized MOF.

**Figure 4:**
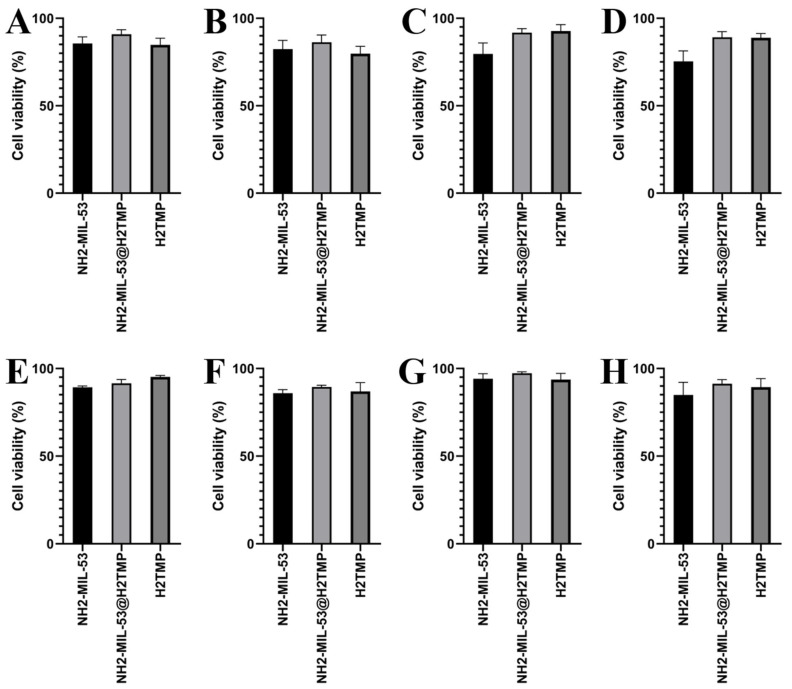
MTT results (with the median values) of the synthesized nanomaterials on the HEK-293 (**A**,**B**), HeLa (**C**,**D**), HepG2 (**E**,**F**), and PC12 (**G**,**H**) after (24 and 48) hours of treatment, respectively. The data shown here are represented by the median values, and the exact concentrations appear in Figure 4.

**Figure 5:**
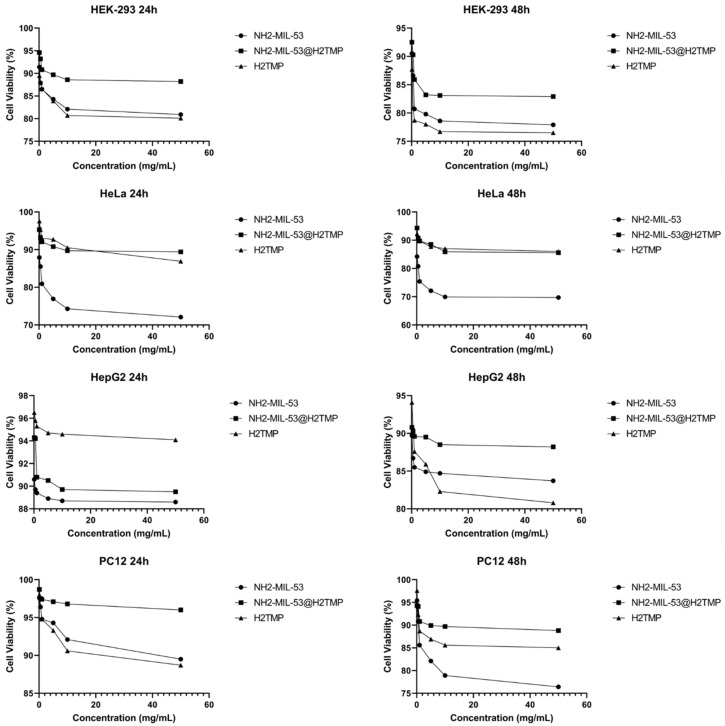
Dose-dependent MTT results of the synthesized nanomaterials.

**Figure 6:**
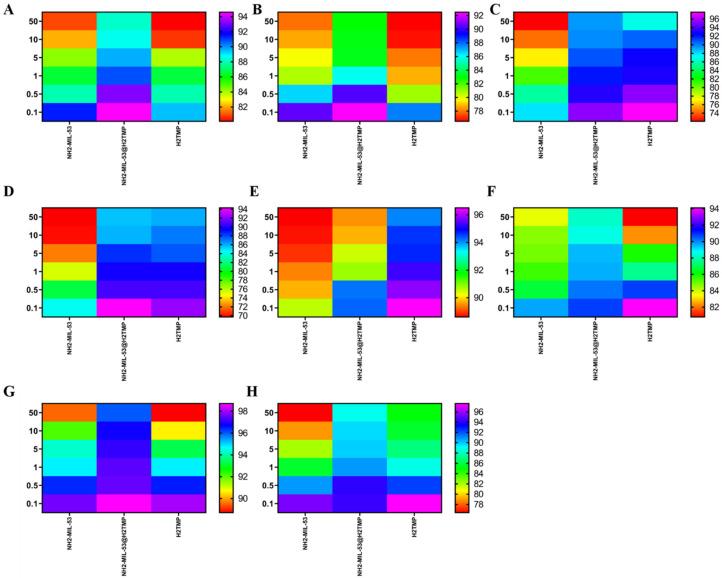
MTT results (dose-dependent with the heat-map shape) of the synthesized nanomaterials on the HEK-293 (**A**,**B**), HeLa (**C**,**D**), HepG2 (**E**,**F**), and PC12 (**G**,**H**) after (24 and 48) hours of treatment, respectively.

After the surface characterizations of the NH_2_-MIL-53@H_2_TMP, the cellular toxicity of these nanomaterials was investigated on the HEK-293, HeLa, HepG2, and PC12 cell lines after 24 and 48 h of treatments. Six different concentrations of the nanomaterials, H_2_TMP, NH_2_-MIL-53, and NH_2_-MIL-53@H_2_TMP, ranging from 0.1 to 50 mg/mL, were investigated. The median cell viability results (Figure 4) showed that most of the synthesized nanomaterials had a relative cell viability of more than 90% in most cases; however, the relative cell viability of the pure MOF was lower than that of porphyrin and the related nanocomposite. Also, the dose-dependent MTT results (Figure 5) indicated that by increasing the concentration of the nanomaterials, the cell viability decreased in all cases. The decreasing rate on the concentrations below 10 mg/mL is much higher than above 10 mg/mL, which is a good result. These types of nanomaterials can be applied as the scaffolds and the implants in order to deliver a specific drug or sensitizer to a specific and targeted tissue, which is mostly the skin. Such nanomaterials can be adjusted to encapsulate a therapeutic and facilitate its release on tumor sites or any targeted cells [57,58,59]. Therefore, they should have significantly high relative cell viability. In the case of photodynamic therapy and photothermal therapy appliances, these types of carriers and/or implants should have relative cell viability of more than 82% on those 4 mentioned cell lines [48,60,61]. For the drug/gene delivery applications, the relative cell viability ideally should be higher than the encapsulated cargo (drug or genetic material); for instance, for targeted delivery of doxorubicin or azithromycin and related therapeutics, the nano-carrier cell viability should be higher than 60% on the HEK-293, MCF-7, HeLa, and PC12 [62,63,64,65]. In this study, the aim of surface modification of the nanomaterials with porphyrin was to increase their sensitivity towards different biomolecules and deployment of the ensuing system for biosensor applications due to the intrinsic feature of the porphyrin to different types of stimuli like hydrogen peroxide and even different metal-free coordinations. Interestingly, the MTT results clearly showed that (Figure 6) by decorating the MOF nanostructures with porphyrin, the relative cell viability increased substantially due to the biomimetic chemistry nature of the porphyrins, same as the hemoglobin and other types of physiological small- and macromolecules, and the final nanoplatforms (or nano-carriers/nanosystems) were turned to a biocompatible form.

**Figure 7:**
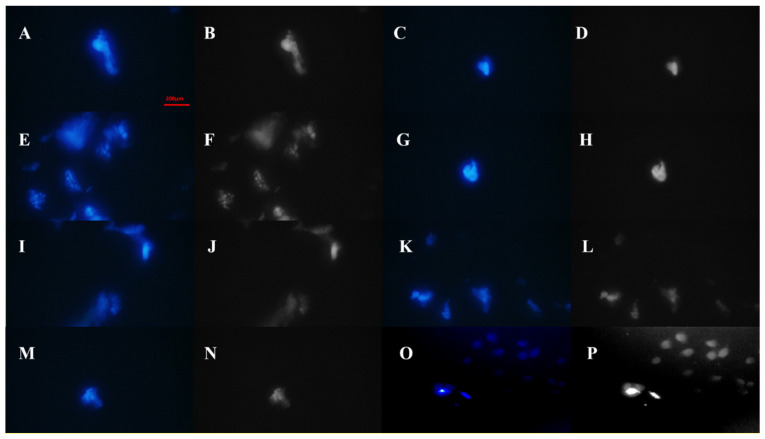
CLSM images of the MCF-7 cell line treated with NH_2_-MIL-53@H_2_TMP (**A**–**N**) after 48 h of treatment and the control group (**O**,**P**). The gray-scale images of the treated DAPI-stained MCF-7 cell lines (**B**,**D**,**F**,**H**,**J**,**L**,**N**,**P**) in order to show the exact morphology of the cells.

Also, in order to determine the possible interactions with the cell membranes and its relationship with the destructive/negative effects on the in vitro cellular experiments, the NH_2_-MIL-53@H_2_TMP was exposed to MCF-7 cell line (stained with 4′,6-diamidino-2-phenylindole (DAPI)) for 48 h, and the morphology of the cells was investigated by confocal laser scanning microscopy (CLSM). The results showed that (Figure 7) the synthesized-decorated MOF does not have any significant cytotoxicity to the cancerous cell line; therefore, the morphology of the stained cells did not change.

**Figure 8:**
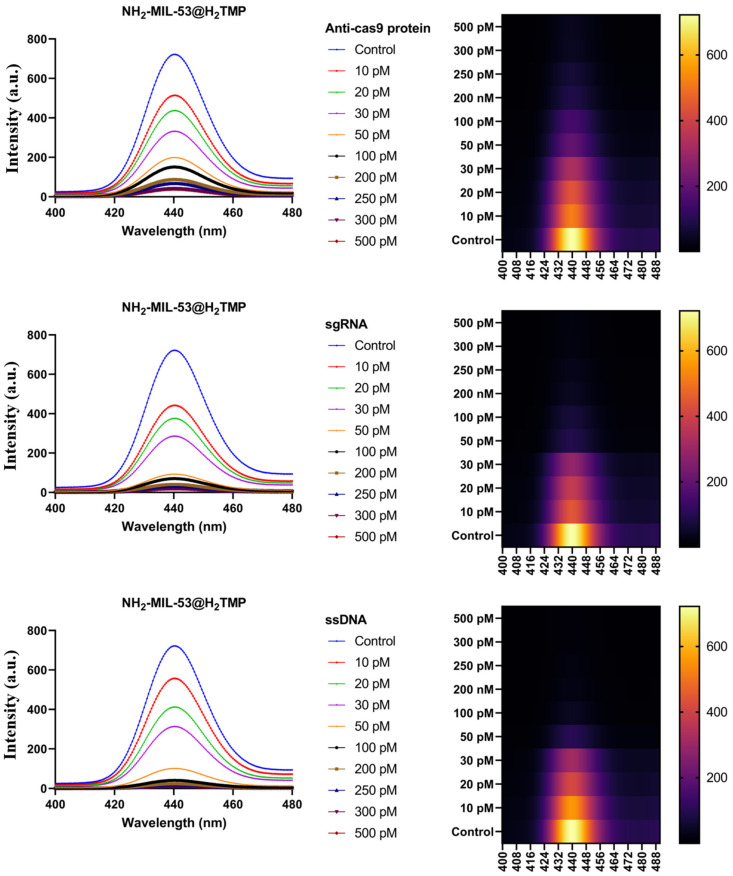
Fluorescence emission spectra of the NH_2_-MIL-53@H_2_TMP in the presence of Anti-cas9 protein, sgRNA, and ssDNA.

In order to determine the possible interactions between the NH_2_-MIL-53@H_2_TMP and different genetic materials and proteins, fluorescence spectroscopy was used. In this context, three different analytes, ssDNA, sgRNA, and Anti-cas9 protein, were selected, and their limit of detection with different concentrations was investigated. Both porphyrin and the MOF are fluorescence active, and they have fluorescence spectra in the range of 400 to 500 nm. However, by coupling these nanostructures via a high-gravity and green approach, a strong fluorescence emission absorbance at around 440 nm was observed (Figure 8). The used porphyrin, H_2_TMP, has a rigid structure; therefore, its conformation is not affected by phase changes and/or solvent changes. In this experiment, the possible interaction and detection ability of the nanomaterial in the presence of those 3 analytes was investigated in the range of 10 to 500 pM of the analytes. Based on the literature, there are limited studies using simple, cost-effective, and tunable nanomaterial to detect trace amounts of those three analytes. Therefore, the detection range of 10 to 500 pM is highly competitive and can be considered as an advantage. By the addition of different concentrations of Anti-cas9 protein to the substrate, the fluorescence spectra decay in a predictable manner with a linear slope; therefore, this detection method can be used for clinical trials. The presence of Anti-cas9 proteins in the samples of animals and/or humans could lead to several mutations as well as severe diseases. Thus, ultrasensitive methods to detect low concentrations of these proteins can help medical doctors for the early diagnosis of different types of mutations and diseases. By the addition of different concentrations of sgRNA and ssDNA, the observed fluorescence decay was different. In the presence of these two analytes, a very fast fluorescence decay after the addition of 30 pM of the analytes was screened, which would be because of the spatial coordination, interactions and/or inhibition around the porphyrin rings [66]. Further, these sequences of genetic materials could fold the whole of the nanostructures, including MOF; therefore, they will affect the MOF spectra as well as the porphyrin at one time. However, all these observations indicate that the NH_2_-MIL-53@H_2_TMP can detect trace concentrations of Anti-cas9 protein, ssDNA, and sgRNA, which is a promising method with easily synthesized, cost-effective and green nanomaterial.

**Figure 9:**
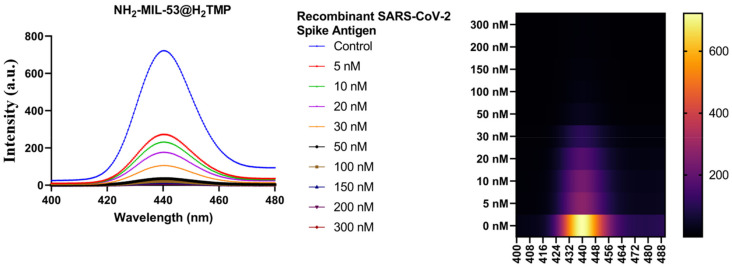
Fluorescence emission spectra of the NH_2_-MIL-53@H_2_TMP in the presence of recombinant SARS-CoV-2 spike antigen.

As mentioned before, the used porphyrin, H_2_TMP, has a rigid structure; therefore, its conformation is not affected by phase changes and/or solvent changes. Therefore, any fluorescent changes in these experiments come from the interactions between them. In this section, the ability of the prepared fluorescent-active nanostructure in detection of low concentrations of recombinant-SARS-CoV-2 spike antigens are investigated (Figure 9). Based on the literature, there are no studies using simple, cost-effective, and tunable nanomaterial to detect trace amounts of recombinant-SARS-CoV-2 spike antigens. Therefore, the detection range of 5 to 300 nM is highly competitive/novel/superior and can be considered an advantage. By the addition of different concentrations of recombinant-SARS-CoV-2 spike antigens to the substrate, the fluorescence spectra decay in a predictable manner with a linear slope; therefore, this detection method can be used for clinical trials.

## 4. Conclusions

Herein, an unprecedented nanocomposite is synthesized based on a green and novel approach by blending the NH_2_-MIL-53 and porphyrins, which were fully characterized. The porphyrins were decorated on the surface of NH_2_-MIL-53 by coating the surface of NH_2_-MIL-53 with leaf extracts, which enabled different types of interactions, including hydrogen bonding, van der Waals, and π-π interactions. The synthesized nanomaterial, NH_2_-MIL-53@H_2_TMP, was applied for the detection of ultra-trace concentrations of ssDNA, sgRNA, anti-cas9 proteins, and recombinant SARS-CoV-2 spike antigen. All of them showed the unprecedented limit of detection of about 10 pM for the genetic materials/proteins and below 10 nM for the recombinant SARS-CoV-2 spike antigen. The potential cytotoxicity of the NH_2_-MIL-53@H_2_TMP and its counterparts was assessed by MTT assay. The results showed that by incorporating the porphyrins on the surface, the nanocomposite was endowed with enhanced biocompatibility and was in a safer format in almost all of the cell lines and at various concentrations; however, in some cases, these differences were too low to be meaningful.

## Data Availability

All data presented in this manuscript as well as complementary information will be available upon request from Navid Rabiee (N.R.); nrabiee94@gmail.com or navid.rabiee@mq.edu.au.

## Supplementary Materials

The following are available online at https://www.mdpi.com/article/10.3390/biom11111714/s1, Figure S1: PXRD pattern for the synthesized MOF, Figure S2: FTIR spectra for the synthesized MOF, Figure S3: Elemental mapping for the synthesized MOF via high-gravity and the use of leaf extracts.

## Sample Availability

Samples of outcomes are available as upon request from Navid Rabiee (N.R.); nrabiee94@gmail.com or navid.rabiee@mq.edu.au.
