# Supplementary material for: Porphyrin Molecules Decorated on Metal-Organic Frameworks for Multi-Functional Biomedical Applications
Source: Biomolecules. 2021 Nov 17;11(11):1714. doi: 10.3390/biom11111714 (PMC8615380; doi:10.3390/biom11111714)
Supplement: Supplementary file 1 [file biomolecules-11-01714-s001.zip › biomolecules-1452242-supplementary.pdf]

Supporting information

Porphyrin molecules decorated on the metal-organic frameworks for multi-functional biomedical application

Synthesis of H_2TMP

Synthesis of the mentioned porphyrin has been performed based on the literature [1–3]. For the synthesis of H_2TMP, 10 mmol mesitaldehyde and 10 mmol distilled pyrrole were added to 100 mL chloroform. The solution was stirred for 10 min as well as purged with the N_2. Then, 3.3 mmol of boron trifluoride-diethyl ether (10^{-3} M) was added to the reaction mixture via a syringe. The solution was stirred at room temperature for 1 hour and oxidized with excess amount of DDQ, followed by the spectrophotometry. At the end, 7.5 mmol of *p*-chloranil was added to the reaction mixture and refluxed for 1 hour. Finally, the reaction mixture was cooled to room temperature and 3.3 mmol of trimethylamine was added followed by removal of solvent on a rotary evaporator. The product was washed for several times by ultrapure water and methanol.

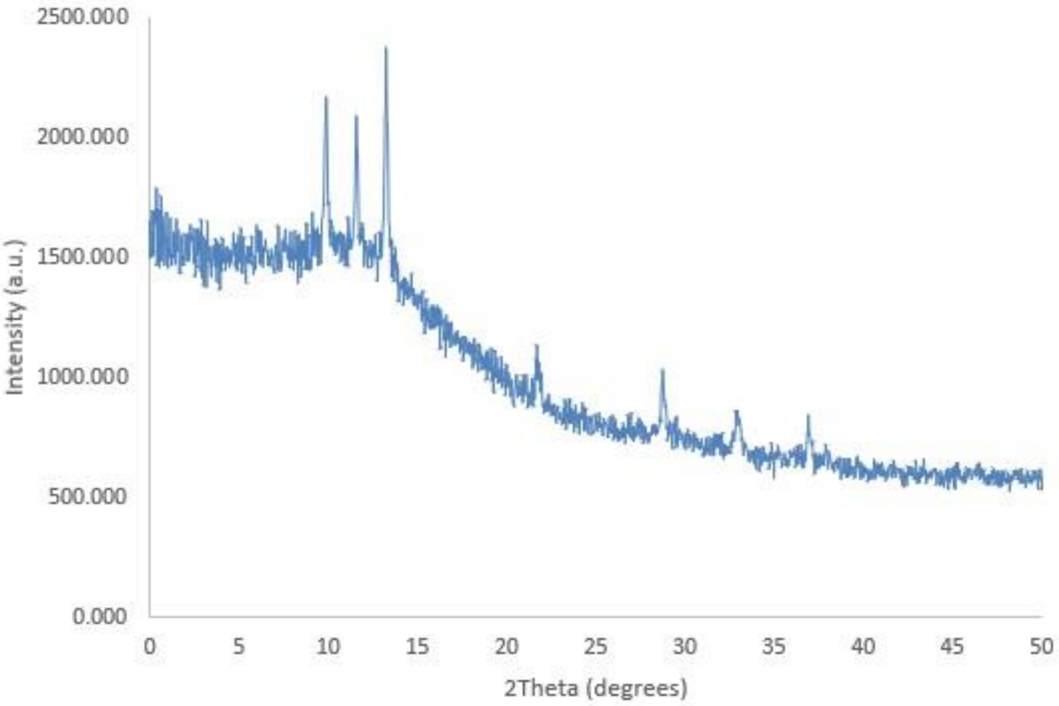

Figure S1. PXRD pattern for the synthesized MOF

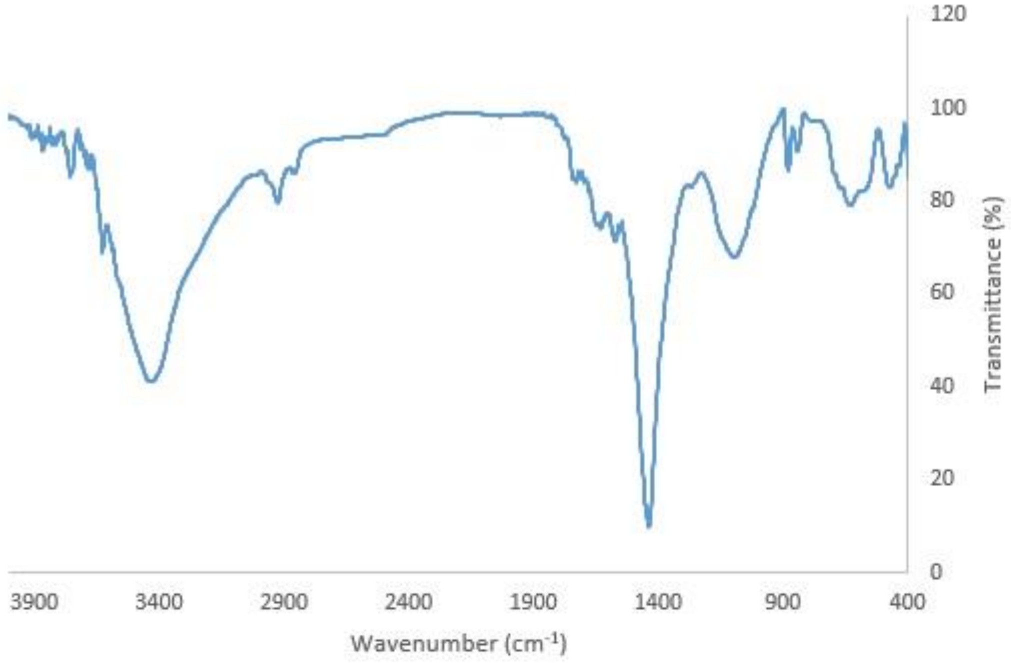

Figure S2. FTIR spectra for the synthesized MOF

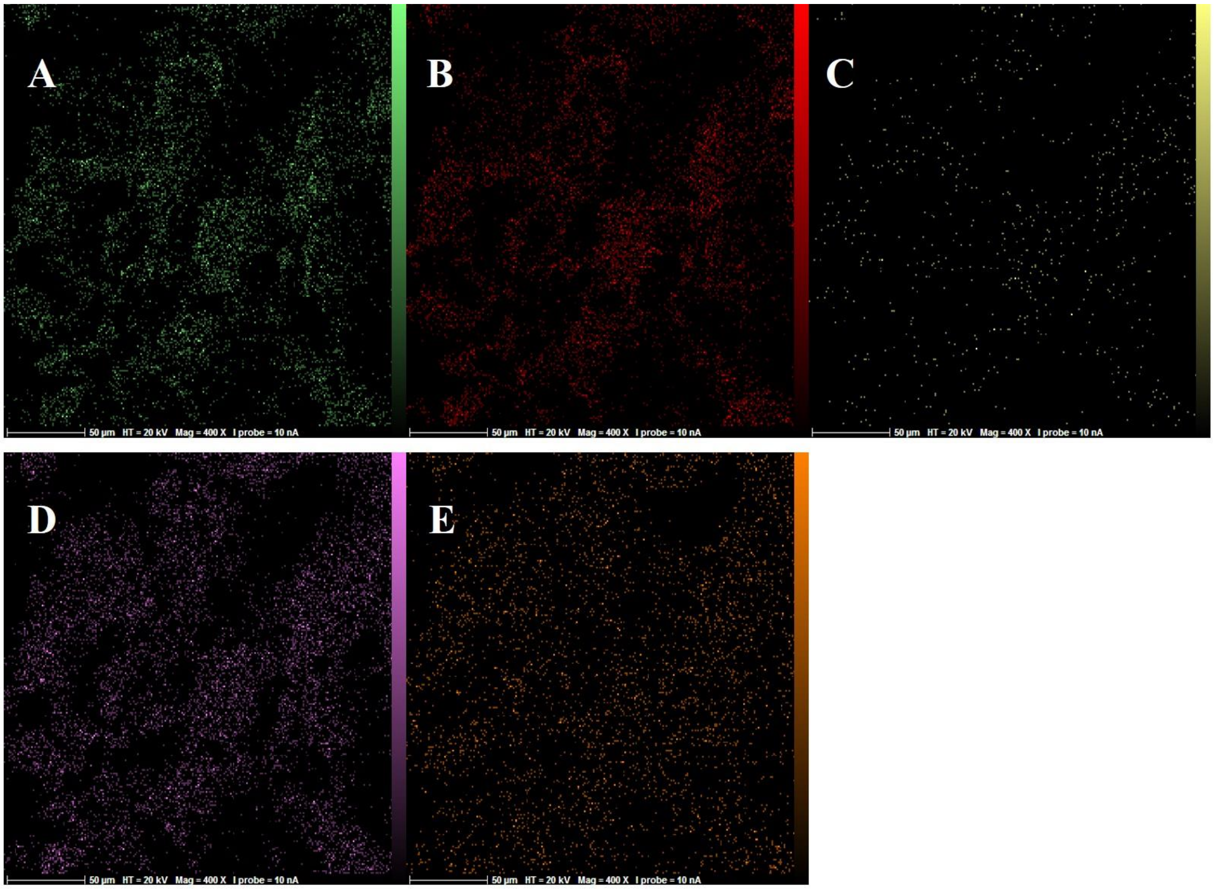

Figure S3. Elemental mapping for the synthesized MOF via high-gravity and the use of leaf extracts; (**A**) Carbon (**B**) Oxygen (**C**) Sulfur (**D**) Chromium and (**E**) nitrogen

(1) Zhou, X.; Tse, M. K.; Wan, T. S.; Chan, K. S. Synthesis of β-mono-, tetra-, and octasubstituted sterically bulky porphyrins via Suzuki cross coupling. *The Journal of organic chemistry* **1996,** *61* (11), 3590-3593.

(2) Rabiee, N.; Bagherzadeh, M.; Ghadiri, A. M.; Fatahi, Y.; Baheiraei, N.; Safarkhani, M.; Aldhaher, A.; Dinarvand, R. Bio-multifunctional noncovalent porphyrin functionalized carbon-based nanocomposite. *Scientific reports* **2021,** *11* (1), 1-15.

(3) Saeb, M. R.; Rabiee, N.; Seidi, F.; Far, B. F.; Bagherzadeh, M.; Lima, E. C.; Rabiee, M. Green CoNi2S4/Porphyrin Decorated Carbon-based Nanocomposites for Genetic Materials Detection. *Journal of Bioresources and Bioproducts* **2021**.
